# The association between socioeconomic status and visual impairments among primary glaucoma: the results from Nationwide Korean National Health Insurance Cohort from 2004 to 2013

**DOI:** 10.1186/s12886-017-0551-y

**Published:** 2017-08-23

**Authors:** Haejune Sung, Hyun Ho Shin, Yunseng Baek, Gyu Ah Kim, Jae Sang Koh, Eun-Cheol Park, Jaeyong Shin

**Affiliations:** 1 Premedical Courses, College of Medicine, Yonsei University, 50, Yonsei-ro, Seodaemun-gu, Seoul, 03722 South Korea; 2 Institute of Vision Research, Department of Ophthalmology, Severance Hospital, College of Medicine, Yonsei University, 50, Yonsei-ro, Seodaemun-gu, Seoul, 03722 South Korea; 3 Institute of Health Services Research, Department of Preventive Medicine, College of Medicine, Yonsei University, 50, Yonsei-ro, Seodaemun-gu, Seoul, 03722 South Korea; 4 Department of Public Health, Graduate School, Yonsei University, 50, Yonsei-ro, Seodaemun-gu, Seoul, 03722 South Korea

**Keywords:** Socioeconomic status, Primary glaucoma, Visual impairment, Income level

## Abstract

**Background:**

Glaucoma is one of the most leading causes of permanent visual impairments in Korea, and social expenses spent for the glaucoma are increasing. This study is to identify association between socioeconomic status and the visual impairments caused by primary glaucoma in Korea.

**Methods:**

This study is based on a cohort study using stratified representative samples in the National Health Insurance claim data from 2002 to 2013 with 1,025,340 representative subjects. Target subjects were patients who are newly diagnosed with primary glaucoma from 2004 to 2013. We conducted a multiple logistic regression analysis depending on the occurrence of visual impairment and its temporal order compared to the glaucoma diagnosis.

**Results:**

Among 1728 patients with primary glaucoma, those with low and middle income shows higher odds ratio (OR) of the visual impairments than those with high income group (low income; OR = 3.42, 95% Confidential Interval (CI):2.06–5.66, middle income; OR = 2.13, 95% CI: 1.28–3.55), in case of the occurrence of the visual impairments preceded the diagnosis of glaucoma.

**Conclusions:**

Glaucoma patients without pre-existing glaucoma history before visual impairment have higher association between socioeconomic status and the occurrence of visual impairments by primary glaucoma. Since glaucoma had not been diagnosed and recognized yet, the differences may have been derived from the disparities of the awareness of the glaucoma. These findings call attention to the correlation between socioeconomic factors and the visual impairments by glaucoma, and raise public health needs over the importance of glaucoma awareness and eye screening for glaucoma, especially for low socioeconomic status.

**Electronic supplementary material:**

The online version of this article (doi:10.1186/s12886-017-0551-y) contains supplementary material, which is available to authorized users.

## Background

Glaucoma is an ophthalmologic disease which is characterized by progressive visual field defect corresponding to excavation of the optic disc. [1, 2] The number of patients who get medical service for glaucoma has been increasing sharply in the Korean society. [3, 4] According to the claim data of Korean National Health Insurance (KNHI) and Health Insurance Review and Assessment Service (HIRA), [5] the number of those who get medical care for glaucoma was 699,463 in 2014, 92.5% increase from 363,329 in 2007.

What is more, severe glaucoma can lead to permanent visual impairments unless it is managed. Based on the WHO Global Data Bank statistics on blindness, [6] about 5.2 million people have blindness caused by glaucoma, which is about 15% of total blindness. Especially in Korea, HIRA data show that glaucoma is the most influential cause of visual impairments among all other diseases including diabetic retinopathy and retinitis. [7] Although it may cause immense loss to respective patients and severe burden to the society, its clinical progress can be controlled after a few examining procedures. [8, 9]

Several factors are known to affect the glaucoma, and socioeconomic status is one of the risk factors for glaucoma. The effects of other factors such as sex and age have also been studied in accordance with the effect of socioeconomic factors. [10] However, few studies have discussed about the socioeconomic status in the risk of visual impairments in developed Asian countries, [11, 12] which have the real importance of studying glaucoma.

Therefore, in this study, we checked the connection between socioeconomic status and the visual impairments caused by primary glaucoma, classifying according to the order of two incidents, a glaucoma diagnosis and an occurrence of visual impairments. In addition, we divided the research population into several subgroups by covariates of the insurance type and researched in the same way.

## Methods

### Data source

We used National Health Information claims data from KNHI database from 2002 to 2013. Korean National Health Information contains cohort data including information on insurance claims for individual medical service, such as patient information, disease code, dates of the service, and the types of medical facilities. [13] These data can represent the whole Korean population since the 1,025,340 subjects included in the data are stratified random sample, and are selected from the total population based on the sex, age, income quintile, region, and health insurance type. Loss of the subjects by censored data is supplemented with newly sampled newborn babies. Based on the representativeness and stability of database, several researches are published using the database. [14, 15] We conducted a cohort study of newly diagnosed glaucoma patients with medication to identify the connection between socioeconomic status and the visual impairment.

### Sample selection

Among all the collected subjects in the database, 1728 with newly diagnosed primary glaucoma (open angle and angle-closure) from 2004 to 2013 were selected. Patients with other types of secondary glaucoma such as trauma, inflammation, and drug-driven glaucoma were excluded. Those who have diseases that can cause the visual impairment from any other cause such as diabetic retinopathy, retinitis, and disorders of vitreous body are also excluded from the selection. We also verified that those patients are newly diagnosed by excluding those who had ever got a medical service for primary glaucoma, recorded from 2002 to 2003 in the National Health Information data. The glaucoma patients who already had visual impairment from 2002 to 2003 are also excluded. Since it requires persistent management to control the progress of glaucoma, selecting those who have 2-year absence of the medical care for glaucoma may assure the first diagnosis.

### Interesting variable

As an indicator of the socioeconomic status, we used an income level. According to the data of National Health Information, income levels are collected on the basis of the amount of monthly insurance premium. The insurance premium is imposed based on salary, real estates, and other financial income. These data are categorized into decile, and include one additional group for those with the lowest income who get medical aid. We reorganized the group into three categories, low income (deciles 1 to 3 and medical aid recipients), middle income (deciles 4 to 6), and high income(deciles 7 to 10) for better analysis. The effect of inflation can be neglected since the income groups are designated by relative proportion.

### Outcome variable

The dependent variable of the study is whether the primary glaucoma patients are diagnosed with visual impairments or not. Ophthalmologists measured the best corrected visual acuity (BCVA) using Snellen chart. In terms of visual fields, it is evaluated by the certified examination such as Humphrey visual field, Goldmann perimetry, and Octopus perimetry.

Then the national government decides the classification of grade based on the severity of visual acuity and available visual field as shown in Table 1 [16, 17]. Visual impairments are designated by visual acuity of eyes with best possible corrections, such as eyeglasses, contact lenses, and surgical treatment. VF is measured by kinetic perimetry. Prominent level VI should be confirmed with retinoscopy, fundus examination, and corneal test. According to the International Classification of Diseases 10th edition, the low vision includes categories 1, 2, and 3 of visual impairment. WHO defines low vision as “a person with low vision is one who has impairment of visual functioning even after treatment and/or standard refractive correction, and has a visual acuity of less than 6/18 to light perception, or a visual field of less than 10 degree from the point of fixation, but who uses, or is potentially able to use, vision for planning and/or execution of a task.” Meanwhile, the current definition of blindness contains either one or both eyes with no perception of light, and less than 3/60 in better eye. Level VI in Korea includes visual acuity impairment and visual field impairment.

**Table 1:** General characteristics of patients with primary glaucoma

Characteristics	N	%
AGE GROUP		
~ 39	310	17.9
40 ~ 49	232	13.4
50 ~ 59	380	22.0
60 ~ 69	431	24.9
70~	375	21.7
SEX		
Male	894	51.7
Female	834	48.3
INCOME GROUP		
High	902	52.2
Middle	490	28.4
Low	336	19.4
Residential Area		
Urban	1323	76.6
Rural	405	23.4
Hospital Level		
General hospital	866	50.1
Hospital Level	141	8.2
Clinic	721	41.7
Insurance Type		
NHI, self-employees	495	28.6
NHI, employees	1169	67.7
Medical Aid	64	3.7
Hypertension		
Normal	942	54.5
Hypertension	786	45.5
Diabetes		0.0
Normal	1341	77.6
Mild Diabetes	255	14.8
Complications	132	7.6
Refractory errors		
No	721	41.7
Yes	1007	58.3
Cataract		
No	882	51.0
Yes	846	49.0
CCI^a^	2.39	±1.97
Total	1728	100

The proportion of those who have a specific type of characteristics is examined among newly diagnosed patients with primary glaucoma from 2004 to 2013

*N* the number of patients examined

^a^mean ± S.D. of CCI (Charlson comorbidity Index): calculated by extracting diabetes and hypertension among comorbidity components

In addition, we supplemented temporal elements between the glaucoma diagnosis and the occurrence of visual impairments; which incident does come prior to the other. Therefore, three different levels of the dependent variable are designated; those who are not diagnosed with visual impairments, those with visual impairments before glaucoma diagnosis, and those with visual impairments after glaucoma diagnosis.

#### Other covariates

Age (−39, 40–49, 50–59, 60–69, 70-), sex, residential area (urban or rural), insurance type (National Health Insurance(NHI) for employees, self-employees, and medical aid recipients), hospital level (general hospital, hospital, clinic), Charlson comorbidity index (CCI) excluding hypertension and diabetes, medical history of hypertension, diabetes, cataract, and refractory error each. Year at diagnosis is also adjusted as a continuous variable.

#### Statistical analysis

To determine the differences in visual impairments among different socioeconomic statuses, we performed a multiple logistic regression analysis. Since stepwise selection is described here, A significance level of 0.1 is required to allow a variable into the model and one of 0.15 to stay in the model. We also examined the full model under consideration of inflated variation in supplementary tables. The group of glaucoma patients without visual impairments was used as the standard group, and each of the group with visual impairment diagnosis before the glaucoma diagnosis, or the group with visual impairment diagnosis after the glaucoma diagnosis was compared to the standard group. All statistical analyses were conducted using SAS 9.4.

## Results

Table 2 displays the characteristics of selected population in Korean patients with Primary Glaucoma. From 2004 to 2013, the number of newly diagnosed patients with primary glaucoma was 1728. The percentage of age group with below 49 was 31.3% while that of age group over 50 was 68.7%. Male recorded higher percentage of total patients, which were 51.7%. Among the patients, 52.2% were classified as high income group, while 28.4% for middle, and 19.4% for low income group.

**Table 2:** Demographic characteristics based on the presence of visual impairment

Variable	Not impaired			Impairment Diagnosed				TOTAL	
				BEFORE Glaucoma		AFTER Glaucoma			
	N	%	N	N	%	N	%	N	*P*-value
Age group									
~ 39	292	94.2	18	8	2.6	10	3.2	310	0.013
40 ~ 49	217	93.5	15	12	5.2	3	1.3	232	
50 ~ 59	357	93.9	23	17	4.5	6	1.6	380	
60 ~ 69	390	90.5	41	31	7.2	10	2.3	431	
70~	330	88.0	45	33	8.8	12	3.2	375	
Sex									
Male	803	89.8	91	66	7.4	25	2.8	894	0.008
Female	783	93.9	51	35	4.2	16	1.9	834	
Income group									
High	850	94.2	52	33	3.7	19	2.1	902	<0.001
Middle	441	90.0	49	33	6.7	16	3.3	490	
Low	295	87.8	41	35	10.4	6	1.8	336	
Residential Area									
Urban	1219	93.6	83	73	5.6	10	0.8	1302	0.568
Rural	367	92.9	28	73	18.5	10	2.5	395	
Hospital Level									
General hospital	796	91.9	70	46	5.3	24	2.8	866	0.612
Hospital	129	91.5	12	8	5.7	4	2.8	141	
Clinic	661	91.7	60	47	6.5	13	1.8	721	
Insurance Type									
NHI, self-employees	456	92.1	39	30	6.1	9	1.8	495	0.011
NHI, employees	1077	92.1	92	61	5.2	31	2.7	1169	
Medical Aid	53	82.8	11	10	15.6	1	1.6	64	
Hypertension									
Normal	876	93.0	66	44	4.7	22	2.3	942	0.073
Hypertension	710	90.3	76	57	7.3	19	2.4	786	
Diabetes									
Normal	1243	92.7	98	65	4.8	33	2.5	1341	<0.001
Mild Diabetes	231	90.6	24	24	9.4	-	0.0	255	
Complications	112	84.8	20	12	9.1	8	6.1	132	
Refractory errors									
No	672	93.2	49	37	5.1	12	1.7	721	0.138
Yes	914	90.8	93	64	6.4	29	2.9	1007	
Cataract									
No	839	95.1	43	26	2.9	17	1.9	882	0.073
Yes	747	88.3	99	75	8.9	24	2.8	846	
CCI^a^	2.81	±2.25		3.10	±2.76	2.27	±2.54		
Total	1586	91.8	142	101	5.8	41	2.4	1728	

*N* the number of patients examined

^a^CCI: Charlson comorbidity index

Table 3 shows demographic characteristics based on the presence of visual impairment. It turns out to be statistically significant in terms of age group (*p* = 0.013), sex (*p* = 0.008), income group (*p* < 0.001), insurance type (*p* = 0.011), diabetes (*p* < 0.001), by the global test.

**Table 3:** multinomial logistic regression (stepwise) for the visual impairment among patients with glaucoma

	Impairment Diagnosed					
	BEFORE Glaucoma			AFTER Glaucoma		
Variable	OR	95% CI	*P*-value	OR	95% CI	*P*-value
Age group						
~ 39	1.00			1.00		
40–49	2.07	(0.82–5.21)	0.123	0.47	(0.12–1.75)	0.259
50–59	1.77	(0.74–4.22)	0.199	0.64	(0.22–1.84)	0.411
60–69	2.87	(1.27–6.49)	0.011	0.95	(0.37–2.42)	0.918
70~	3.96	(1.74–9.02)	0.001	1.97	(0.78–4.91)	0.148
Sex						
Female	1.00					
Male	2.17	(1.40–3.34)	0.001	1.65	(0.85–3.18)	0.136
Income level						
High	1.00			1.00		
Middle	2.13	(1.28–3.55)	0.003	1.53	(0.75–3.09)	0.233
Low	3.42	(2.06–5.66)	<.001	1.08	(0.41–2.80)	0.870
Diabetes						
Normal	1.00			1.00		
Mild Diabetes	1.64	(0.97–2.75)	0.064			
Complications	1.79	(0.91–3.48)	0.089	3.11	(1.31–7.34)	0.010
Refractory errors						
No						
Yes	1.39	(0.91–2.14)	0.130	2.18	(1.07–4.41)	0.030
Year at diagnosis						
a year	0.92	(0.85–0.99)	0.022	0.74	(0.654–0.83)	<.001

The result of multivariate analysis on visual impairment among newly diagnosed patients with primary glaucoma based on multinomial logistic regression model with stepwise process

*OR* odds ratio

Multivariate analysis on odds ratio(OR) of visual impairment among the patients with primary glaucoma is shown in Table 4. We used stepwise selection for preventing inflation of variance. In case of the patients diagnosed with primary glaucoma after the occurrence of the visual impairment, both low income group (OR = 2.13, 95% CI: 1.28–3.55, *p* < 0.001) and middle income group (OR = 2.13, 95% CI: 1,28–3.55, *p* = 0.003) have higher odds ratio of the visual impairment, compared to the high income group. We did not find statistical significance in case of the patients diagnosed with primary glaucoma before the occurrence of the visual impairment in terms of the differences in income group.

**Table 4:** Multivariate analysis on visual impairment of newly diagnosed patients with primary glaucoma by the type of National Health Insurance (NHI)

	BEFORE Glaucoma			AFTER Glaucoma		
Income Group	OR	95% CI	*P*-value	OR	95% CI	*P*-value
Self-employee of NHI						
High	1.00			1.00		
Middle	2.72	(1.03–1.17)	0.002	1.16	(0.23–5.83)	0.546
Low	4.95	(1.79–13.69)	0.042	1.73	(0.29–10.28)	0.850
Employee of NHI						
High	1.00			1.00		
Middle	1.98	(1.07–3.65)	0.029	1.85	(0.83–4.15)	0.133
Low	2.38	(1.21–4.69)	0.022	0.85	(0.23–3.10)	0.801

The result of multivariate analysis on visual impairment of newly diagnosed patients with primary glaucoma classified by the type of National Health Insurance (NHI). The patients who are self-employee of NHI and employee of NHI are examined separately.

Increased age is related to the higher odds ratio for visual impairment among particiants with previous visual impairment before glaucoma diagnosis. Considering the factors of diabetes, glaucoma patients with diabetes unaccompanied by complications (OR = 1.79, 95% CI: 0.97–3.48, *p* = 0.089) shows higher odds ratio of the impairment in case of the diagnosis of glaucoma followed after the occurrence of visual impairment, although it is statistically insignificant slightly. Interestingly, the presence of refractory error is related to the visual impairment after the diagnosis of glaucoma. (OR = 2.18, CI: 1.07–4.41, *p* = 0.030) We also performed the full model under consideration of variance inflation (Additional file 1: Table S1).

**Fig. 1:**
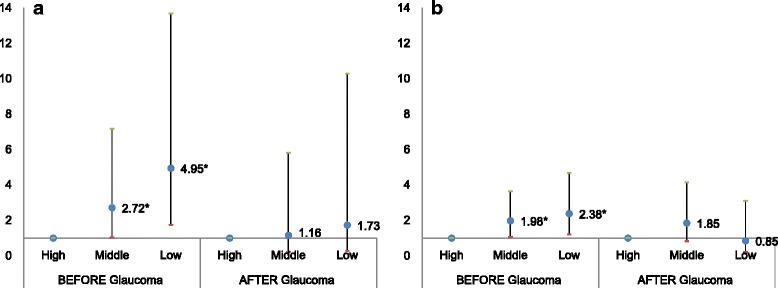
Multinomial logistic regression analysis based on the National Health Insurance type, **a** Self-employees and **b** Employees. Compared to the high income group, middle and low income group show higher odds ratios for visual impairment before the first diagnosis of glaucoma, in both self-employees and employees. However, the association is stronger in self-employees than in employees

Sensitivity analysis on OR of visual impairment between Korean self-employees and employees with primary glaucoma is presented in Fig. 1. Based on information of insurance types collected from KHNI, patients are classified into two different groups of insurance types, self-employees and employees. With each of the insurance type groups, we conducted a multivariate analysis. As a result, in case of the impairment preceding the glaucoma diagnosis, differences in the impairment were statistically significant for self-employees of middle (OR = 2.72, 95% CI: 1.03–7.18, *P* = 0.002) and low income group (OR = 4.95, 95% CI: 1.79–13.69, *p* = 0.042) compared to high income group. In contrast, there was no significance for the income group of self-employees in case of the impairment followed after glaucoma diagnosis. In case of the impairment antedating the glaucoma diagnosis, it was significant for employees of middle (OR = 1.98, 95% CI: 1.07–3.65, *p* = 0.029) and low income group (OR = 2.38, 95% CI: 1.21–4.69, *p* = 0.022), compared to high income group. However, there is no significance for the income group of employees in case of the impairment going after the glaucoma diagnosis.

## Discussion

Throughout this study, we determined the effect of socioeconomic status on the risk of visual impairment in patients with primary glaucoma in a cohort study using stratified random samples from Korean Health Information collected by KNHI. As a result, the effect of socioeconomic status on the risk of impairment was significant only when the occurrence of the visual impairment preceded the diagnosis of glaucoma. Primary glaucoma patients with lower income had significantly increased visual impairment when they had not diagnosed with the disease. Multivariate analysis with subgroups divided into two different groups of insurance type also showed the same aspect of the trend.

Glaucoma is considered to have less noticeable symptoms that the patients with it can hardly recognize. [18, 19] Even though glaucoma is substantially worsened, central vision is known to be maintained relatively longer. Among different types of primary glaucoma, chronic open-angle glaucoma is hard to notice due to the adaptation of nerves to high intraocular pressure, and the symptoms of acute closed-angle glaucoma such as headache and nausea are easily confused with other diseases. Normal-tension glaucoma, another type of primary glaucoma, is accompanied by relatively low intraocular pressure, which makes it even hard to find out. [20]

It is crucial to figure out how socioeconomic status affects the visual impairments among glaucoma patients. There are possibilities that the lack of awareness of glaucoma as serious disease may influence low detection rate. Basically, there is low awareness of glaucoma in Korean population. According to Korea National Health and Nutrition Examination Survey (KNHANES), only 9% of glaucoma patients are aware of their diseases. [3, 10] In addition, patients with lower economic status have even less perception of glaucoma. According to the cross-sectional study in US, less affluent and less educated group of people has lower frequency of visiting eye care provider. [21] Accordingly, people with low socioeconomic status and low frequency of using medical services have increased impairments of closed-angle glaucoma. The accessibility of using medical services affects the detection of glaucoma, and that accessibility is influenced by socioeconomic status including income and education. [21, 22] Not only detection but also severity of glaucoma can be affected by socioeconomic factors. [23] Another study has examined the relationship between socioeconomic status and the ratio of severe glaucoma to total glaucoma, and glaucoma patients with serious economic poverty has increased proportion of severe one. [23–25] This strongly supports our findings since severe glaucoma can lead to visual loss if it is neglected.

However, there is no significant connection between the visual impairment and the income in case of glaucoma patients who have already been diagnosed. Since the cost for glaucoma treatment is relatively less compared to healthcare for other eye conditions [26] and the universal health coverage in Korea cover the medical cost almost [27], the patients with low income can also use proper medical care for glaucoma preventing it from worsening. Moreover, we did not find any significance in the relationship between the impairment and the residential area, or the size of medical facility. Glaucoma is kind of a life-long disease that needs to be continuously managed, and in general, the excessive intraocular pressure can be controlled by medication.

Interestingly, we found the significance of mild diabetes in case of the occurrence of impairments that preceded the prevalence of glaucoma. Those glaucoma patients with mild diabetes had higher OR of the occurrence of visual impairments if their glaucoma was not checked. It is unclear whether the result of significance in glaucoma is a driver or a passenger factor of the occurrence of impairments. If the diabetes acts as a driver factor of the occurrence of impairments, diabetes may be the confounding variable that mediates the hidden relationship between socioeconomic status and the difference in the severity of the glaucoma. In case of presuming the diabetes as a passenger factor of the impairments, we can predict that poor concerns about health care and low frequency of using medical facilities may both influence patients’ lack of recognition of glaucoma and diabetes that they have. There are a few studies that concern to the diabetes as a risk factor for glaucoma [28–30], but the causal relationship has not been proved clearly. Further analysis on the effect of diabetes on glaucoma is needed for lucid explanation.

Before drawing conclusion from the results, the limitation of this study should be discussed. First, considering low detection of glaucoma, which is about 1.5–2% in Korea [3, 10] and those patients having glaucoma concurrently with visual impairments, the absolute amounts of selected samples are not that a lot. Second, we could not keep track of patients with glaucoma without symptoms. Those patients may not feel necessity to use medical service, thus their information would not be collected and included in the database. Similarly, some of the patients with visual impairments may not visit a clinic or a hospital to be diagnosed, and the information of their impairments would not also be included in the data either. Third, Korean National Health Information claim data we used do not contain the information of medical records so we did not determine the severity of glaucoma of each patient. Thus, we cannot completely neglect the possibility that the severity of glaucoma may differ between income groups even if there was no significance of visual impairment found in the case of the glaucoma that has already been diagnosed. Fourth, there are still unmeasured confounders or unadjusted medical conditions, related to visual impairment. To overcome this shortcoming, we excluded diabetic retinopathy, retinitis, disorders of vitreous body, and aging macular degeneration, which are the most common diseases causing visual impairment in Korea. The history of cataract and refractory errors are adjusted for multiple linear regression analysis. Fifth, it is not able to consider age as a continuous variable. For de-identification in database, the Korean government provides this public data with some limitations. As part of these, the data do not include the exact birth date. Rather, it describes the age intervals. Although there is still some variance in the same category, we have adjusted the age group of ten years interval.

In spite of the limitations, there are several strengths in our study. First, this study was the first to examine the socioeconomic effects on the visual impairment by applying temporal order of the incidents. There were many studies using cross-sectional data to identify the correlation [31], but none of them classified samples into two different cases of the time order using cohort data. Since our study showed different aspects between different cases of time order, it would be much meaningful to understand the influence of socioeconomic status on the visual impairments by glaucoma. Second, our study not only dealt with the glaucoma but also focused on the visual impairments. Since glaucoma needs to be persistently managed, and visual impairment itself is more direct issue that threats people’s quality of life, our study approached more to practical matters; the accessibility on the examination of glaucoma should also be enhanced as the developed clinical techniques in treatment. Third, we excluded patients with other diseases that can cause visual impairments so we can verify that occurred visual impairments in the samples are solely driven by glaucoma.

## Conclusions

In case of patients with primary glaucoma after visual impairment, those with lower income are more vulnerable to the occurrence of visual impairments. Since disparities of the visual impairments by glaucoma are only detected when the glaucoma had not been diagnosed and recognized yet, the differences may have been derived from the disparities of the awareness of the glaucoma. These findings call attention to the correlation between socioeconomic factors and the visual impairments by glaucoma, and raise public health needs over the importance of glaucoma awareness and early eye screening for glaucoma.

## Additional file

Additional file 1: Table S1. Multinomial logistic regression analysis under full model. The adjusted odds ratio using multinomial logistic regression under full model in consideration of variance inflation. (DOCX 18 kb)

## Abbreviations

CCI: Charlson Comorbidity Index
CI: Confidential Interval
HIRA: Health Insurance Review and Assessment Service
KNHANES: Korea National Health and Nutrition Examination Survey
KNHI: Korean National Health Insurance
OR: Odds ratio
