# Supplementary material for: The association between socioeconomic status and visual impairments among primary glaucoma: the results from Nationwide Korean National Health Insurance Cohort from 2004 to 2013
Source: BMC Ophthalmol. 2017 Aug 23;17:153. doi: 10.1186/s12886-017-0551-y (PMC5569511; doi:10.1186/s12886-017-0551-y)
Supplement: Additional file 1: Table S1. — Multinomial logistic regression analysis under full model. The adjusted odds ratio using multinomial logistic regression under full model in consideration of variance inflation. (DOCX 18 kb) [file 12886_2017_551_MOESM1_ESM.docx]

|  | **Impairment Diagnosed** | | | | | | |
| --- | --- | --- | --- | --- | --- | --- | --- |
|  | BEFORE Glaucoma diagnosis | | |  | AFTER Glaucoma diagnosis | | |
| **Variable** | OR | 95% CI | P-VALUE |  | OR | 95% CI | P-VALUE |
| **AGE GROUP** |  |  |  |  |  |  |  |
| ~39 | 1.00 |  |  |  | 1.00 |  |  |
| 40-49 | 2.00 | (0.79-5.07) | 0.143 |  | 0.50 | (0.13-1.92) | 0.315 |
| 50-59 | 1.77 | (0.72-4.30) | 0.210 |  | 0.69 | (0.22-2.07) | 0.505 |
| 60-69 | 2.85 | (1.21-6.67) | 0.016 |  | 1.09 | (0.39-3.03) | 0.863 |
| 70~ | 3.97 | (1.64-9.57) | 0.002 |  | 2.49 | (0.87-7.05) | 0.086 |
| **SEX** |  |  |  |  |  |  |  |
| Female | 1.00 |  |  |  |  |  |  |
| Male | 2.17 | (1.40-3.36) | 0.001 |  | 1.61 | (0.82-3.12) | 0.105 |
| **INCOME GROUP** |  |  |  |  |  |  |  |
| High | 1.00 |  |  |  | 1.00 |  |  |
| Middle | 2.09 | (1.25-3.48) | 0.005 |  | 1.62 | (0.78-3.31) | 0.190 |
| Low | 2.87 | (1.64-4.98) | 0.000 |  | 0.99 | (0.35-2.76) | 0.976 |
| **Residential Area** |  |  |  |  |  |  |  |
| Urban | 1.00 |  |  |  | 1.00 |  |  |
| Rural | 0.96 | (0.59-1.55) | 0.862 |  | 0.94 | (0.47-2.26) | 0.941 |
| **Hospital Level** |  |  |  |  |  |  |  |
| General hospital | 1.00 |  |  |  | 1.00 |  |  |
| Hospital Level | 1.11 | (0.70-1.73) | 0.655 |  | 0.66 | (0.32-1.36) | 0.266 |
| Clinic | 1.04 | (0.46-2.32) | 0.925 |  | 1.70 | (0.53-5.376) | 0.368 |
| **Insurance Type** |  |  |  |  |  |  |  |
| NHI, employees | 1.00 |  |  |  | 1.00 |  |  |
| NHI, self-employees | 0.96 | (0.60-1.53) | 0.865 |  | 1.52 | (0.69-3.36) | 0.296 |
| Medical Aid | 1.91 | (0.78-4.68) | 0.157 |  | 2.25 | (0.22-23.26) | 0.495 |
| **Year at diagnosis** |  |  |  |  |  |  |  |
| **Per year** | 0.92 | (0.84-0.99) | 0.033 |  |  |  |  |
| **CCI†** |  |  |  |  | 0.82 | (0.66-1.01) | 0.065 |
| **Per score** | 0.94 | (0.83-1.05) | 0.285 |  |  |  |  |
| **Hypertension** |  |  |  |  |  |  |  |
| Normal | 1.00 |  |  |  | 1.00 |  |  |
| Hypertension | 1.08 | (0.67-1.74) | 0.752 |  | 1.20 | (0.54-2.65) | 0.645 |
| **Diabetes** |  |  |  |  |  |  |  |
| Normal | 1.00 |  |  |  | 1.00 |  |  |
| Mild Diabetes | 1.67 | (0.98-2.83) | 0.058 |  |  |  |  |
| Complications | 1.80 | (0.89-3.60) | 0.097 |  | 2.91 | (1.15-7.342) | 0.024 |
| **Refractory error** |  |  |  |  |  |  |  |
| No | 1.00 |  |  |  | 1.00 |  |  |
| Yes | 1.49 | (0.95-2.31) | 0.078 |  | 2.42 | (1.17-4.97) | 0.017 |
| **Cataract** |  |  |  |  |  |  |  |
| No | 1.00 |  |  |  |  |  |  |
| Yes | 2.82 | (1.16-4.86) | 0.002 |  | 1.17 | (0.53-2.59) | 0.701 |
